# Effect of combined physical–cognitive training on the functional and cognitive capacity of older people with mild cognitive impairment: a randomized controlled trial

**DOI:** 10.1186/s12916-024-03469-x

**Published:** 2024-07-08

**Authors:** Yolanda Castellote-Caballero, María del Carmen Carcelén Fraile, Agustín Aibar-Almazán, Diego Fernando Afanador-Restrepo, Ana María González-Martín

**Affiliations:** 1 Department of Health Sciences, Faculty of Health Sciences, University of Jaén, Jaén, 23071 Spain; 2 Department of Health Sciences, Faculty of Health Sciences, University of Atlántico Medio, Las Palmas de Gran Canaria, 35017 Spain; 3 Department of Education and Psychology, Faculty of Social Sciences, University of Atlántico Medio, Las Palmas de Gran Canaria, 35017 Spain; 4 Faculty of Health Sciences and Sport, University Foundation of the Área Andina-Pereira, Pereira, 660004 Colombia; 5 Department of Psychology, Centro de Educación Superior de Enseñanza e Investigación Educativa, Plaza de San Martín, 4, Madrid, 28013 Spain

**Keywords:** Mild cognitive impairment, Combined training, Cognitive ability, Physical health

## Abstract

**Background:**

The increase in population aging highlights the growing prevalence of mild cognitive impairment, prompting the adoption of interventions that combine physical exercise and cognitive training to improve health and cognitive performance in older adults. The aim of this study was to analyze the efficacy of a combined program on physical and cognitive health in older people with cognitive impairment.

**Methods:**

A 12-week randomized controlled clinical trial involving 95 participants (aged 72.12 ± 4.25 years), 47 individuals participated in a control group (CG) that only underwent cognitive stimulation, while 48 individuals were in an experimental group (EG) that participated in a combined program. Balance was measured using the Tinetti scale, upper body strength was assessed with the arm curl test, lower body strength was evaluated with the 30-s chair stand test, flexibility was tested using the back scratch test and chair sit-and-reach test, physical function was measured with the Timed Up and Go test, cognitive function was assessed using the Mini Mental State Examination, cognitive impairment was evaluated with the Montreal Cognitive Assessment, verbal fluency was tested with the Isaac test, and executive functions were assessed using the Trail Making Test.

**Results:**

The results of the study show significant improvements in both physical and cognitive aspects, such as balance, gait, upper and lower body strength, flexibility, physical function, cognitive function, cognitive impairment, verbal fluency, and executive functions in the group that carried out the intervention compared to the control group.

**Conclusion:**

A combined program for older individuals with mild cognitive impairment leads to enhancements in physical and cognitive health. These improvements underscore the importance of integrating physical exercise with cognitive training as an effective strategy for enhancing overall health and quality of life in older adults.

**Trial registration:**

NCT05503641.

## Background

Population aging is an increasingly notable phenomenon, driven by an increase in life expectancy and a decrease in birth rates [1]. In 2020, it was observed that 22.9% of Spain’s population was over 65 years old. Projections suggest that by the middle of the twenty-first century, approximately 31.4% of the Spanish population will be in this age group, with 11.6% of them being octogenarians [2]. Given these projections and the concerning annual incidence rate of 7.7 million new dementia diagnoses, expected to rise to over 135.5 million by 2050 [3], there is a critical need to implement effective and cost-efficient interventions to curb the increase in new dementia cases. It is crucial to direct efforts towards older adults displaying signs of initial cognitive impairment, such as mild cognitive impairment (MCI) [4].

MCI represents an intermediate stage between normal cognitive aging and the early stages of dementia. It is characterized by a mild reduction in cognitive abilities associated with aging, without significantly impacting daily activities [5]. Over the past decade, there has been considerable interest in investigating cognitive and neural changes in individuals with MCI, as this group is identified as being at high risk of progressing to Alzheimer’s disease (AD) [6, 7]. Certainly, the focus of research on MCI has expanded beyond memory deficits to include deficits not directly related to memory, particularly emphasizing executive functions [8]. This shift in research focus is crucial as problems in executive functions have been identified as potential aggravators of memory deficits and as playing a pivotal role in the progression from MCI to more severe forms of dementia [9]. Verbal fluency ability has been shown to decline in individuals several years before they meet the diagnostic criteria for MCI or dementia [10]. Therefore, verbal fluency tests have been recognized as effective tools for distinguishing between individuals with normal cognitive function and those who are starting to experience cognitive impairment [11].

MCI extends beyond affecting only cognitive abilities and is closely linked to several physical health problems [12]. There is growing evidence suggesting that physical characteristics, such as muscle weakness and slower walking speed, may serve as effective biomarkers to anticipate the onset of cognitive decline [13–15]. A relationship has been observed between variations in the execution of motor tasks, such as grip strength and gait pattern, and changes in cognitive capacity, pointing to these motor changes as early indicators of possible cognitive impairment [16]. This phenomenon can be attributed to several factors. On the one hand, both cognitive and motor capacity rely on the nervous system to perform physical activities, implying that any impairment in this system can affect both cognition and motor skills [13] On the other hand, hyperintensities in the white matter, a frequent neuropathological sign in MCI, have been linked to a reduction in muscle mass [17] and decreased walking speed [18]. In addition, physical exercise, by improving muscle fitness, may play a crucial role in preserving neuronal health. This suggests that lifestyle acts as a compensatory element in the face of cognitive decline and physical weakness [19].

Management tactics for MCI include both pharmacological and nonpharmacological approaches, such as physical exercise, cognitive therapies, and psychological support [20]. Prevailing evidence suggests prioritizing nonpharmacological strategies in the treatment of MCI to avoid and minimize the adverse effects associated with the use of medications [21]. Among these, intervention with physical exercise has demonstrated a beneficial effect in preventing and managing cognitive impairment in older adults, presenting advantages such as fewer side effects and greater adherence compared to pharmacological treatments [22]. It is crucial to highlight the importance of regularity and continuity in physical training, as these factors are essential to improve cognitive performance in individuals with MCI. Furthermore, integrating cognitive tasks during exercise may intensify these positive benefits, underscoring the synergy between physical activity and cognitive stimulation in this population [23].

The integration of a physical training program with specific cognitive training, known as combined training, could significantly increase the likelihood of cognitive benefits [24]. A meta-analysis by Karssemeijer et al. [25] supports the efficacy of these combined interventions, pointing to benefits in activities of daily living and cognitive status in older adults with MCI or dementia, underscoring the clinical importance of a training approach that combines physical and cognitive exercises. This type of intervention has been previously implemented in other populations, such as healthy older adults [26], stroke patients [27], or patients with Parkinson’s disease [28] where the evidence shows significant improvement in gait through combined training.

However, it is important to note that psychomotor programs, commonly used in clinical practice, especially for people with mental health and/or cognitive impairment, have not been widely included or evaluated in the context of larger research [29]. This gap in the literature suggests the need to further explore the potential of psychomotor programs in conjunction with cognitive stimulation interventions in older adults. Therefore, the aim of this study is to analyze the effects of a combined training program on physical and cognitive health in older adults with MCI. This objective is based on the hypothesis that combined training has better effects on balance, strength, flexibility, physical and cognitive function, verbal fluency, and executive functions compared to just cognitive stimulation.

## Methods

### Study design

This study is a randomized controlled trial aimed at analyzing the effects of a combined training program on physical and cognitive health in older adults with MCI (NCT05503641). All participants provided informed consent before the study began, following the Declaration of Helsinki, good practices, and applicable laws and regulations. The study has received approval from the Ethics Committee of the University of Jaén (MAR.22/8.TFM).

### Participants

**Fig. 1:**
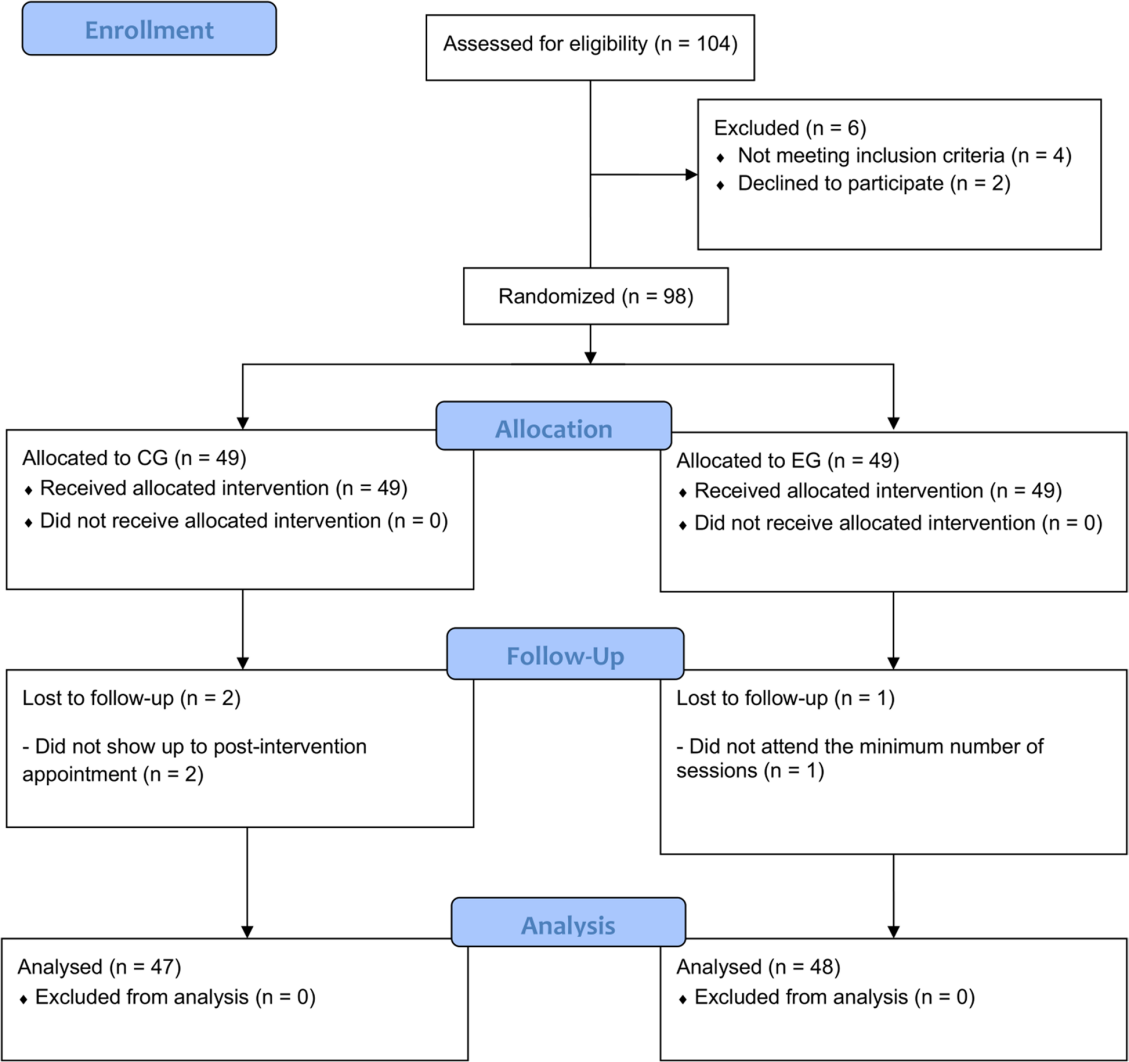
Flowchart

From a total of 104 participants who were initially contacted, 98 met the inclusion criteria and agreed to participate in the study (Fig. 1). Participants for this study were recruited through social media advertisements and informational posters at local community centers frequented by people potentially interested in exercise and cognitive training programs. Each participant received a detailed description of the study and was asked about her interest in participating. Those who expressed initial interest were invited to an information session where the study requirements, expected benefits, and possible risks were discussed in detail. During this session, each participant was also assessed for eligibility to ensure they met the study-specific inclusion criteria. To be able to participate, individuals had to (i) be men and women over 65 years of age, who voluntarily agreed to participate, and who did not participate in any additional physical exercise program; (ii) present mild cognitive impairment, confirmed by a score less than 25 on the Mini-Mental State Examination, administered by a trained professional; and (iii) be able to understand instructions and respond to questionnaires designed for this study, as well as participate in the established physical tests. Exclusion criteria were (i) having visual problems that cannot be corrected with the use of glasses, contact lenses, or surgery. This includes conditions such as advanced macular degeneration, progressive diabetic retinopathy, advanced glaucoma, and other ocular pathologies that significantly limit central or peripheral vision and (ii) be enrolled in another physical exercise program for the duration of the study.

### Randomization

Participants who met the inclusion criteria were randomly assigned to an experimental group (EG) consisting of a total of 49 participants and a control group (CG) with 49 individuals. This randomization was carried out using a computer-produced random number table. Subsequently, sealed opaque envelopes were used, and group assignment was performed by an independent investigator unrelated to participant selection, intervention, or data collection.

### Intervention

The combined program in the study consisted of two interrelated parts:

A Cognitive stimulation sessions aimed at maintaining and/or improving cognition. Each session was organized in groups of up to 8 people; it was carried out 4 days a week, with each person having the opportunity to participate in two weekly sessions of between 45 and 50 min, carried out in the morning. The activities developed were the following: (i) Memory: 1. Recall exercises: use of memory cards with images and words to exercise visual and verbal memory. 2. Story sequencing: participants order sequences of images or texts that tell a short story to improve sequential memory and comprehension; (ii) Language: 1. Word games: exercises such as word searches and rhyming games to stimulate verbal fluency and vocabulary. 2. Narration and description: activities that involve describing objects or situations in detail or constructing stories in groups; (iii) Executive functions: 1. Puzzles and logical problems: puzzles, Sudoku, and simple mathematical problems to stimulate reasoning and planning. 2. Classification and categorization exercises: organize objects according to categories, promoting organization, and decision-making; (iv) Attention and concentration: 1. Sustained attention exercises: tasks such as following patterns on a light board or maintaining concentration on a reading while recording certain stimuli. 2. Divided attention games: activities that require handling two tasks simultaneously, such as counting objects while answering questions; (v) Perception: 1. Recognition of shapes and colors: use of building blocks or cards to identify shapes and colors. 2. Spatial perception exercises: tasks that involve estimating distances or identifying changes in the configuration of objects in space. For this cognitive stimulation program, memory cards were used, including images and words for memory exercises; board games, such as puzzles, word games and puzzles, which serve to promote reasoning and planning; digital tools, including tablets and computers that offer interactive exercises adjustable in difficulty level; and writing and drawing materials to support activities that involve narration and description. These materials were selected for their ability to adapt to the specific needs of participants, allowing for personalization of interventions to optimize cognitive outcomes. All participants in both groups performed the same cognitive stimulation activities, adapted to their cognitive and sensory abilities.
B Psychomotor sessions are designed to maintain and/or improve the motor, cognitive, social, and affective functioning of the participants. These last two due to group interaction that encourages communication and interaction between participants, overcoming challenges and emotional regulation through relaxation and body awareness activities. All of this is achieved through the following activities: (i) postural tone through chair exercises such as seated heel raises and chair torso twists, slow walks, wall exercises, and gentle stretches; (ii) body scheme through tactile recognition exercises to improve body awareness and imitation of shapes; (iii) laterality with ball passing exercises and crossed movements; (iv) fine and gross motor skills such as stringing beads and agility circuits; (v) balance through the performance of balance postures, walking in place, weight balancing, and line walking; (vi) flexibility through arm–shoulder stretches, shoulder stretches, and wrist and ankle rotations. Two weekly sessions were held, each lasting 45 to 50 min. The sessions were structured in three parts: presentation of the activity (5 min) in which an introduction and explanation of the activity is given, ensuring that participants understand the objectives and the steps to follow; carrying out the activity (30–40 min) where the active execution of the tasks designed to promote psychomotor and cognitive skills occurs; and return to calm (5 min) in which cooling and relaxation activities will be carried out to stabilize the physical and mental state of the participants at the end of the session. All activities were adapted to the individual abilities of the participants and to this end their complexity was adjusted, ergonomic adaptations were made through chairs or mats, and regular breaks were integrated to avoid fatigue.

### Outcomes and measurement instruments

Data were collected both before and immediately after the end of the intervention phase. Descriptive details such as gender, age, educational level, marital status, and employment status were obtained through self-administered surveys supervised by experienced interviewers. Height was measured using an Asimed T201-T4 adult stadiometer, and weight was measured with a Tefal digital scale with precision from 100 g to 130 kg, allowing the calculation of the body mass index (BMI) as the ratio between weight in kilograms and height in meters squared.

#### Balance

The Tinetti scale was used to measure the physical variables of balance, gait, and risk of falling [30, 31]. This scale is divided into two parts: one that measures static and dynamic balance with 9 items and a maximum score of 17 points and another part that measures gait with 7 items and a maximum score of 12 points. The sum of the two parts is used to evaluate the risk of falling, with a higher score indicating a lower risk of falling. A score between 19 and 24 points indicates a risk of falling, while a score below 19 indicates a low risk.

#### Upper body strength

The arm curl test [32] was used to count the number of arm curls completed in 30 s on the right and left sides using a dumbbell. Participants began the test seated in a standard 17-in chair, keeping one arm upright at the side of the chair. Progressively, participants rotated the palm upward as they flexed the arm through the full range of motion and then fully extended it to return to the starting position. For the test to be considered valid, the participant’s arm flexion had to be completing, moving from a fully flexed position to a fully extended position at the elbow.

#### Lower body strength

The 30-s chair stand test [32] was performed as the initial assessment in the battery to evaluate lower limb muscle strength, counting the number of repetitions completed in 30 s. Participants began seated at the edge of a standard 17-in-high chair with their arms crossed in front of their chest. They were instructed to perform as many sit-to-stand cycles as possible at their maximum speed. Guidance was provided to ensure that participants stood up fully on each repetition and touched the seat of the chair on the way down.

#### Flexibility

To evaluate functional flexibility, the back scratch test (BST) [33] was conducted for the upper extremities and the chair sit-and-reach test (CSRT) [34] for the lower extremities. The BST assessed shoulder joint flexibility by having participants stand and place one hand behind their neck, moving it down the spine, while the other hand was placed on the lower back and moved up the spine. This process was repeated with the opposite arms/hands, and the distance between the tips of the middle fingers of both hands was measured. If the fingers touched, “0” was marked; if not, the distance (in cm) was measured with negative ( −) values indicating a gap and positive ( +) values indicating overlap. For the CSRT, participants sat in a chair against a wall for stability and tried to touch their right and left toes. If they reached only the fingers, the score was “0”. Reaches beyond the toes were recorded as negative values ( −), while positive values ( +) were assigned to those who could reach further (in cm).

#### Physical function

Physical function was evaluated by measuring travel speed using the Timed Up and Go (TUG) test [35]. This test involves standing up from a chair, walking a distance of 3 m as quickly as safely possible, turning around and sitting down again. Generally, a cone or some other clear marking is placed at the 3-m point to indicate where participants should turn. The time recorded during the TUG test was converted into an estimate of travel speed using the formula [6/ (TUG time)] × 1.62. A value of ≤ 0.8 m/s was considered the standard limit for slow travel speed [36].

#### Cognitive function

To assess overall cognitive function, the Mini-Mental State Examination (MMSE) [37], a widely recognized tool for detecting potential severe cognitive impairment, was utilized. This test evaluates five key areas of cognition: attention, calculation, orientation, memory, and language. The maximum achievable score on the MMSE is 30 points, indicating better overall cognitive performance. The established benchmark scores are as follows: a score of 27 or above is considered normal, scores of 24 or above may indicate possible cognitive issues, a score between 12 and 24 suggests impaired cognitive function, and a score ranging from 9 to 12 is associated with symptoms of dementia.

#### Cognitive impairment

The Montreal Cognitive Assessment (MoCA) is a concise and comprehensive test consisting of 12 components designed to evaluate seven different cognitive areas: visuospatial and executive skills (including tasks such as tracing a figure, copying a cube, and drawing a clock), naming, attention, the ability to recall number sequences in reverse order, sustained attention ability (assessed by a constant touch exercise), language skills (including sentence repetition and verbal fluency), abstraction ability (assessed through a verbal task), immediate and delayed memory, and orientation. The maximum possible score on the MoCA is 30 points, with values equal to or greater than 26 indicative of normal cognitive functioning [38]. It is important to note that the MoCA includes a scoring adjustment based on the individual’s educational level: an extra point is added to the total score for those participants who have received 12 years of education or less. This adjustment aims to compensate for the potential influences of educational level on test outcomes, providing a more equitable assessment of cognitive abilities across diverse population groups.

#### Verbal fluency

To assess verbal fluency, the Isaac test was administered. In this test, participants are required to list as many words as possible within a semantic category (e.g., animals, fruits, cities, or colors) within 60 s. Each category allows for a maximum score of 10 points, resulting in a total possible score of 40 points. A higher score indicates greater verbal fluency ability [39].

#### Executive functions

The Trail Making Test (TMT) was utilized to evaluate executive function, emphasizing tasks that demand motor coordination and visual skills within time constraints. This assessment comprises two parts: the first, TMT-A, evaluates attention and speed by connecting numbered circles sequentially; the second, TMT-B, involves connecting circles alternating between letters and numbers, specifically targeting executive function [40]. A longer time taken to complete the test was interpreted as less efficient performance in this context.

### Sample size calculation

Considering the size of the minimum detectable difference between the groups, the level of significance and the desired statistical power. In our scenario, we assume a mean difference of 0.7 units [38] between the groups in the variables associated with the strength of upper and lower limbs, a significance level of 5% (corresponding to a z value of 1.96), and a power of 90% (corresponding to a *z* value of 1.28). There were 98 participants, distributed equally between the control and experimental groups; however, an additional 15% was considered due to loss during follow-up.

### Statistical analysis

All statistical analyses were performed with the SPSS statistical program, version 20.0, for Windows (SPSS, Inc., Chicago, IL). Statistical significance was determined at *P* < 0.05. The results of this study were presented as the means and standard deviations for continuous variables, and frequencies and percentages for categorical variables. The Kolmogorov–Smirnov test was used to assess the normality of the data distribution. Student’s *t* and chi-square tests were used for the continuous and categorical variables, respectively, to determine the possible differences between both study groups before the study began. A mixed analysis of variance was carried out to analyze any differences in values between the studied variables, in which the study group was considered the inter-group factor (CG vs EG), and the measurement time of the variables (pre- and post-intervention) the intra-group factor. The dependent variables were balance (the Tinetti scale), upper body strength (the arm curl test), lower body strength (the 30-s chair stand test), flexibility (BST and CSRT), physical function (TUG test), cognitive function (MMSE), cognitive impairment (MoCA), verbal fluency (Isaac test), and executive functions (TMT). All analyses were carried out independently for each dependent variable and the possible interactions “group × measurement time” was analyzed. Cohen’s *d* statistic was used to assess the effect size of possible inter-group and intra-group differences. Values < 0.2 indicate an insignificant effect size, between ≥ 0.2 and < 0.5—small, between ≥ 0.5 and < 0.8—medium, and ≥ 0.8—large.

## Results

Table 1 shows the baseline characteristics of the participants at the beginning of the study, in which it can be seen that there were no significant differences between the groups. All participants completed at least 91.4% of the sessions and no injuries or adverse effects were reported during the course of the intervention.

**Table 1:** Baseline characteristics of study participants

		**Total** **(*n* = 95)**	**EG** **(*n* = 48)**	**CG** **(*n* = 47)**	***P*-value**
Age (years)		72.12 ± 4.25	71.85 ± 3.70	72.38 ± 4.78	0.640
Sex	Male	68 (71.6)	35 (36.8)	33 (34.7)	0.565
	Female	27 (28.4)	13 (13.7)	14 (14.7)	
Marital status (%)	Single	19 (20.0)	8 (8.4)	11 (11.6)	0.062
	Married	45 (47.4)	27 (28.4)	18 (18.9)	
	Separated/widowed	31 (32.6)	13 (13.7)	18 (18.9)	
Education	No formal education	37 (38.9)	24 (25.3)	13 (13.7)	0.967
	Primary education	47 (49.5)	23 (24.2)	24 (25.3)	
	Secondary education	11 (11.6)	1 (1.1)	10 (10.5)	
Employment status	Retired	63 (66.3)	32 (33.7)	31 (32.6)	0.304
	Employed	5 (5.3)	1 (1.1)	4 (4.2)	
	Unemployed	27 (28.4)	15 (15.8)	12 (12.6)	
Weight (kg)		69.61 ± 13.46	71.28 ± 13.88	67.89 ± 12.95	0.625
Height (m)		1.64 ± 0.13	1.64 ± 0.13	1.63 ± 0.14	0.449
BMI (kg/m^2^)		25.74 ± 2.39	26.34 ± 2.72	25.13 ± 1.81	0.164
Balance		8.83 ± 3.7	9.25 ± 3.25	8.40 ± 4.01	0.167
Gait		7.96 ± 2.68	7.85 ± 2.26	8.06 ± 3.07	0.080
Fall Risk		16.79 ± 5.18	17.10 ± 4.90	16.47 ± 5.49	0.280
Upper body strength		19.44 ± 3.25	19.77 ± 3.08	19.11 ± 3.42	0.200
Lower body strength		15.96 ± 2.76	16.21 ± 2.66	15.70 ± 2.87	0.618
Flexibility right arm		− 11.28 ± 10.91	− 12.58 ± 9.87	− 9.96 ± 11.84	0.186
Flexibility left arm		− 13.92 ± 10.73	− 15.13 ± 9.50	− 12.68 ± 11.83	0.082
Flexibility right leg		− 7.46 ± 9.15	− 7.96 ± 9.90	− 6.96 ± 8.40	0.185
Flexibility left leg		− 5.94 ± 8.82	− 6.75 ± 9.42	− 5.11 ± 8.17	0.242
Physical Function		10.73 ± 1.52	10.97 ± 1.64	10.49 ± 1.37	0.547
MMSE		21.64 ± 3.88	22.17 ± 4.21	21.11 ± 3.48	0.200
MoCA		21.40 ± 1.06	21.38 ± 1.06	21.43 ± 1.06	0.992
Verbal Fluency		25.65 ± 2.43	25.77 ± 2.48	25.53 ± 2.40	0.530
Executive Functions part A		108.75 ± 45.57	114.42 ± 39.60	102.96 ± 50.73	0.195
Executive Functions part B		195.82 ± 76.49	229.29 ± 56.39	161.64 ± 79.65	0.061

Data are expressed as mean and standard deviation and frequency and percentage for continuous or categorical variables respectively

* EG* Experimental group, *CG* Control group, *MMSE* Mini Mental State Examination, *BMI* Body mass index, *MoCA* Montreal Cognitive Assessment

### Balance

According to our findings, in balance, the results showed that the main effect of time was significant, *F*[93] = 13.956, *p* = 0.000, *η*^2^ = 0.130, indicating significant differences over time. The main effect of the group was not statistically significant, *F*(93) = 3.255, *p* = 0.074, *η*^2^ = 0.034. However, the group × time interaction was significant, *F*(93) = 4.592, *p* = 0.035, *η*^2^ = 0.047, suggesting that changes over time varied significantly between groups, indicating an increase in balance and therefore, an improvement in the experimental group. Statistically significant differences were found between pre- and post measurement in the treatment/training group:* t* (47) =  − 3.455, *p* = 0.001, Cohen’s *d* = 0.33, and statistically significant differences between both groups in the postintervention measurement: *t* (93) = 2.477, *p* = 0.015, Cohen’s *d* = 0.51. Regarding gait, in the analysis of variance conducted to examine the effects of group and the interaction between group and time, the results showed no statistically significant differences in the main effects of the group, *F*(93) = 0.637, *p* = 0.427, *η*^2^ = 0.007, or in the main effects of time, *F*(93) = 0.396, *p* = 0.531, *η*^2^ = 0.004. However, the interaction between group × time was significant, *F*(93) = 11.836, *p* = 0.001, *η*^2^ = 0.113, indicating significant differences in how the groups varied over time, indicating an increase in gait quality in the experimental group. Finally, with respect to the total score of the Tinetti scale referring to the risk of falls, the results showed that the main effect of time was significant, *F*(93) = 8.920, *p* = 0.004, *η*^2^ = 0.088, indicating significant differences over time. The main effect of the group was not statistically significant, *F*(93) = 2.784, *p* = 0.099, *η*^2^ = 0.029. However, the group × time interaction was significant, *F*(93) = 14.628, *p* = 0.000, *η*^2^ = 0.136, suggesting that changes over time varied significantly between groups, indicating a higher score on the EG suggesting a lower risk of falls. Statistically significant differences were found between the pre- and post measurement in the treatment/training group: *t* (47) = 2.800, *p* = 0.006, Cohen’s *d* = 0.39, and statistically significant differences between both groups in the postintervention measurement: *t* (93) = 4.212, *p* = 0.000, Cohen’s *d* = 0.57 (Table 2).

**Table 2:** Effects of a psychomotor and cognitive stimulation program on physical capacity

	EG (*n* = 26)		CG (*n* = 24)		Group			Time			Group × Time		
	Pre	Post	Pre	Post	*F*(93)	*p*-value	*η*^2^	*F*(93)	*p*-value	*η*^2^	*F*(93)	*p*-value	*η*^2^
Tinetti balance	9.25 ± 3.25	10.27 ± 2.83	8.40 ± 4.01	8.68 ± 3.41	3.255	0.074	0.034	13.956	0.000	0.130	4.592	0.035	0.047
Tinetti gait	7.85 ± 2.26	8.56 ± 1.74	8.06 ± 3.07	7.57 ± 2.83	0.637	0.427	0.007	0.396	0.531	0.004	11.836	0.001	0.113
Tinetti fall risk	17.10 ± 4.90	18.83 ± 4.00	16.47 ± 5.49	16.26 ± 4.93	2.784	0.099	0.029	8.920	0.004	0.088	14.628	0.000	0.136
Upper body strength	19.77 ± 3.08	18.54 ± 3.25	19.11 ± 3.42	19.89 ± 3.23	0.334	0.565	0.004	0.547	0.461	0.006	11.390	0.001	0.109
Upper body strength	16.21 ± 2.66	14.21 ± 2.74	15.70 ± 2.87	16.17 ± 2.31	2.192	0.142	0.023	10.759	0.001	0.104	27.928	0.000	0.231
Flexibility right arm	− 12.58 ± 9.87	− 8.15 ± 6.68	− 9.96 ± 11.84	− 13.96 ± 16.05	0.588	0.445	0.006	0.036	0.851	0.000	13.284	0.000	0.125
Flexibility left arm	− 15.13 ± 9.50	− 8.75 ± 6.48	− 12.68 ± 11.83	− 13.36 ± 11.93	0.285	0.594	0.003	35.145	0.000	0.274	53.964	0.000	0.367
Flexibility right leg	− 7.96 ± 9.90	− 2.81 ± 7.90	− 6.96 ± 8.40	− 7.38 ± 9.03	1.153	0.286	0.012	14.706	0.002	0.102	14.706	0.000	0.137
Flexibility left leg	− 6.75 ± 9.42	− 3.19 ± 6.94	− 5.11 ± 8.17	6.57 ± 8.68	0.294	0.589	0.003	3.080	0.083	0.032	17.768	0.000	0.160
Physical Function	10.97 ± 1.64	10.27 ± 1.47	10.49 ± 1.37	10.93 ± 1.50	0.103	0.749	0.001	0.621	0.433	0.007	12.889	0.001	0.122

Quantitative variables are presented as mean and standard deviation

*EG* Experimental group, *C *Eontrol group

### Upper body strength

In the upper body strength results, an analysis of variance (ANOVA) was performed to evaluate the effects of group, time as well as the interaction between group and time. The results indicated that the main effect of group was not statistically significant, *F*(93) = 0.334, *p* = 0.565, *η*^2^ = 0.004, nor was the main effect of group *F*(93) = 0.547, *p* = 0.461, *η*^2^ = 0.006. However, the interaction between group × time showed statistical significance, *F*(93) = 11.390, *p* = 0.001, *η*^2^ = 0.109, suggests that the treatment had a positive effect in this regard. Statistically significant differences were observed between pre- and post measurement in the treatment/training group: *t* (47) = 3.474, *p* = 0.001, Cohen’s *d* = 0.39, and statistically significant differences between both groups in the post-intervention measurement, *t* (93) =  − 2.035, *p* = 0.045, Cohen’s *d* = 0.42 (Table 2).

### Lower body strength

In the lower body strength results, an analysis of variance (ANOVA) was conducted to assess the effects of group, time, and the group-by-time interaction. The results showed that the main effect of time was significant, *F*(93) = 10.759, *p* = 0.001, *η*^2^ = 0.104, indicating significant differences over time. The main effect of the group was not statistically significant, *F*(93) = 2.192, *p* = 0.142, *η*^2^ = 0.023. However, the group × time interaction was significant, *F*(93) = 27.928, *p* < 0.001, *η*^2^ = 0.231, suggesting that changes over time varied significantly between groups, indicating an increase in lower body strength. Statistically significant differences were observed between pre- and post measurement in the treatment/training group: *t* (47) = 16.793, *p* = 0.000, Cohen’s *d* = 0.74, and statistically significant differences between both groups in the postintervention measurement: *t* (93) =  − 3.769, *p* = 0.000, Cohen’s *d* = 0.77 (Table 2).

### Flexibility

In the right arm, the results did not show statistically significant differences in the main effects of the group, *F*(93) = 0.588, *p* = 0.445, *η*^2^ = 0.006, or in time, *F*(93) = 0.036, *p* = 0.851, *η*^2^ = 0.000. However, the interaction between group × time was significant, *F*(93) = 13.284, *p* < 0.001, *η*^2^ = 0.125, indicating significant differences in how the groups varied over time. Statistically significant differences could be observed between the pre- and post measurement in the treatment/training group: *t* (47) =  − 6.178, *p* = 0.000, Cohen’s *d* = 0.53, and statistically significant differences between both groups in the postintervention measurement: *t* (93) = 2.313, *p* = 0.023, Cohen’s *d* = 0.47. In the left arm, the results showed that the main effect of time was significant, *F*(93) = 35.145, *p* = 0.000, *η*^2^ = 0.274, indicating significant differences over time. The main effect of the group was not statistically significant, *F*(93) = 0.285, *p* = 0.594, *η*^2^ = 0.003. However, the group × time interaction was significant, *F*(93) = 53.964, *p* = 0.000, *η*^2^ = 0.367, suggesting that changes over time varied significantly between groups. Statistically significant differences were observed between the pre- and post measurement in the treatment/training group: *t* (47) =  − 7.927, *p* = 0.000, Cohen’s *d* = 0.78, and statistically significant differences between both groups in the postintervention measurement: *t* (93) = 2.348, *p* = 0.021, Cohen’s *d* = 0.48. Regarding the right leg, the results showed that the main effect of time was significant, *F*(93) = 14.706, *p* = 0.002, *η*^2^ = 0.102, indicating significant differences over time. The main effect of the group was not statistically significant, *F*(93) = 1.153, *p* = 0.286, *η*^2^ = 0.012. However, the group × time interaction was significant, *F*(93) = 14.706, *p* = 0.000, *η*^2^ = 0.137, suggesting that changes over time varied significantly between groups. The results showed statistically significant differences between pre- and post measurement in the treatment/training group: *t* (47) =  − 5.974, *p* = 0.000, Cohen’s *d* = 0.58, and statistically significant differences between both groups in the postintervention measurement: *t* (93) = 2.628, *p* = 0.010, Cohen’s *d* = 0.54. Finally, in the left leg, the results did not show statistically significant differences in the main effects of the group, *F*(93) = 0.294, *p* = 0.589, *η*^2^ = 0.003, or in time, *F*(93) = 3.080, *p* = 0.089, *η*^2^ = 0.032. However, the interaction between group × time was significant, *F*(93) = 17.768, *p* < 0.001, *η*^2^ = 0.160, indicating significant differences in how the groups varied over time. Statistically significant differences were found between pre- and post measurement in the treatment/training group: *t* (47) =  − 4.661, *p* = 0.000, Cohen’s *d* = 0.43, and statistically significant differences between both groups in the postintervention measurement: *t* (93) = 2.103, *p* = 0.038, Cohen’s *d* = 0.44 (Table 2). All these results suggested that the participants who carried out the combined training obtained lower scores than at the beginning of the study, suggesting greater flexibility.

### Physical function

Regarding physical function, the results did not show statistically significant differences in the main effects of the group, *F*(93) = 0.103, *p* = 0.749, *η*^2^ = 0.001, or in time, *F*(93) = 0.621, *p* = 0.433, *η*^2^ = 0.007. However, the interaction between group × time was significant, *F*(93) = 12.889, *p* = 0.001, *η*^2^ = 0.122, indicating significant differences in how the groups varied over time. The experimental group obtained higher results, suggesting an improvement in their physical function. The results showed statistically significant differences between pre- and post measurement in the treatment/training group: *t* (47) = 3.564, *p* = 0.001, Cohen’s *d* = 0.45, and statistically significant differences between both groups in the post-intervention measurement: *t* (93) = 2.103, *p* = 0.038, Cohen’s *d* = 0.03 (Table 2).

### Cognitive function

According to our findings, in cognitive function, the results showed that the main effect of time was significant, *F*(93) = 26.290, *p* = 0.000, *η*^2^ = 0.044, indicating significant differences over time. The main effect of the group was not statistically significant, F(93) = 3.175, *p* = 0.078, *η*^2^ = 0.033. However, the group × time interaction was significant, *F*(93) = 4.297, *p* = 0.041, *η*^2^ = 0.044, suggesting that changes over time varied significantly between groups, indicating an improvement in general cognitive function. Statistically significant differences were found between pre- and post measurement in the treatment/training group: *t* (47) =  − 5.663, *p* = 0.000, Cohen’s *d* = 0.33, and statistically significant differences between both groups in the post-intervention measurement: *t* (93) = 2.103, *p* = 0.038, Cohen’s *d* = 0.43 (Table 3).

### Cognitive impairment

In cognitive impairment, the results showed that the main effect of time was significant, *F*(93) = 14.307, *p* = 0.000, *η*^2^ = 0.126, indicating significant differences over time. The main effect of the group was statistically significant, *F*(93) = 13.428, *p* = 0.000, *η*^2^ = 0.126 and the group × time interaction was significant, *F*(93) = 26.213, *p* = 0.000, *η*^2^ = 0.321, suggesting that changes over time varied significantly between groups. This indicates that the experimental group obtained higher results, so significant improvements are observed after the intervention. Statistically significant differences were found between the pre- and post measurement in the treatment/training group: *t* (47) =  − 8.690, *p* = 0.000, Cohen’s *d* = 1.22, and statistically significant differences between both groups in the postintervention measurement: *t* (93) = 5.252, *p* = 0.000, Cohen’s *d* = 1.32 (Table 3).

### Verbal fluency

Regarding verbal fluency, the results showed that the main effect of time was significant, *F*(93) = 5.678, *p* = 0.019, *η*^2^ = 0.058, indicating significant differences over time. The main effect of the group was statistically significant, *F*(93) = 8.645, *p* = 0.004, *η*^2^ = 0.085 and the group × time interaction was significant, *F*(93) = 33.569, *p* = 0.000, *η*^2^ = 0.265, suggesting that changes over time varied significantly between groups, suggesting more pronounced improvements in the treatment group because they obtained lower scores compared to the beginning of the study. Statistically significant differences were observed between the pre- and post measurement in the treatment/training group: *t* (47) =  − 7.490, *p* = 0.000, Cohen’s *d* = 0.64, and statistically significant differences between both groups in the postintervention measurement: *t* (93) = 6.479, *p* = 0.000, Cohen’s *d* = 1.08 (Table 3).

### Executive functions

Finally, regarding part A of the Trial Making Test, the results did not show statistically significant differences in the main effects of the group, *F*(93) = 0.165, *p* = 0.685, *η*^2^ = 0.002, or in time, *F*(93) = 1.055, *p* = 0.307, *η*^2^ = 0.011. However, the interaction between group × time was significant, *F*(93) = 23.424, *p* = 0.000, *η*^2^ = 0.201, indicating significant differences in how the groups varied over time. As for part B, the results showed that the main effect of group was significant, *F*(93) = 14.435, *p* = 0.000, *η*^2^ = 0.134, indicating significant differences over time. The main effect of the time was not statistically significant, *F*(93) = 3.228, *p* = 0.076, *η*^2^ = 0.034. However, the group × time interaction was significant, *F*(93) = 15.775, *p* = 0.000, *η*^2^ = 0.145, suggesting that changes over time varied significantly between groups. There were statistically significant differences between the pre- and post measurement in the treatment/training group: *t* (47) = 4.104, *p* = 0.000, Cohen’s *d* = 0.56, and statistically significant differences between both groups in the postintervention measurement: *t* (93) = 2.006, *p* = 0.048, Cohen’s *d* = 0.41 (Table 3). In both parts of the test, the experimental group obtained lower scores, which indicates that this group observed improvements.

## Discussion

The main objective of this study was to analyze the effects of a combined training program on physical and cognitive health in older adults with MCI. After a 12-week intervention consisting of a combination of cognitive training and psychomotor exercises with a frequency of two times per week and a compliance rate of over 91.4%, improvements in the physical and cognitive health of older adults with MCI were evidenced, mainly in terms of strength, flexibility, balance, physical function, cognition, verbal fluency, and executive functions [41].

It has been shown that both performing cognitive training alone and performing physical exercise as the only training have beneficial effects in healthy older adults and those with mild cognitive impairment, as in the systematic review by Zhang et al. [42], in which they demonstrated that cognitive training was a viable strategy to improve cognitive function in older adults with cognitive impairment and the study by Rivas-Campo [43], who managed to obtain significant improvements in different cognitive variables through an intervention of high intensity functional training in older adults with cognitive impairment. On the other hand, multidomain interventions, defined as such because they are composed of two or more interventions, may have even greater benefits than physical or cognitive exercise alone. The findings of the present study demonstrated that when combining cognitive training with physical exercise, significant differences were found in the intergroup comparison, which favored the intervention/training group, with a small effect size (*t* (93) = 2.103, *p* = 0.038, Cohen’s *d* = 0.43). Consistent with our results, Vaughan et al. [43] observed that after 60 min of multicomponent training per week, improvements could be obtained in the Stroop test and the Trail Making Test, but unlike ours, with healthy older adults. Like a recent systematic review [44], associations were observed between multidomain interventions compared to single interventions in older adults with cognitive impairment.

The effects of physical exercise on cognition have been extensively investigated [45, 46] attributing the improvement to enhancements in brain metabolism, brain structure, brain connectivity, cerebral vascular function, and brain plasticity [47–50]. In the case of motor training, it has been observed to induce different changes compared to those caused by aerobic exercise. Aerobic exercise enhances cognition through improvements in cardiorespiratory fitness, whereas motor exercise directly influences cognitive processes. Additionally, aerobic training appears to affect neuroplasticity broadly, while motor training, being task-oriented, impacts neuroplasticity specifically [51].

Multiple studies have established a connection between cognition and physical performance, particularly highlighting balance as one of the physical abilities that deteriorates rapidly most rapidly when cognitive ability is impaired [52]. In a recent study, Xiao et al. [53] identified a correlation between cognition and balance in middle-aged and older adults. They argued that this correlation stems from the competition for limited central attentional resources during posture maintenance and dual-task performance. This is particularly relevant as older adults, due to aging, tend to allocate greater cognitive resources to task performance [54]. Similar to the findings on balance [55], combined interventions involving motor and cognitive training have demonstrated effectiveness in enhancing gait parameters, such as step length and gait speed, especially in populations affected by conditions like multiple sclerosis [56]. Likewise, in a clinical trial [57], it was observed that older adults with mild cognitive impairment who received aerobic resistance exercises with computerized sequential cognitive training significantly improved cognition, although some results were inconsistent. Similarly, one study [58] examined the effectiveness of dual-task cognitive training, physical exercise, and a combination of both trainings on dual-task performance, but concluded that future studies are needed that compare the effects of sequential physical and cognitive training and combined to better understand the extent of the synergistic effects of these interventions on cognition in older adults [59].

Our study, consistent with the aforementioned findings, demonstrated that a 12-week combined intervention in older adults with MCI led to improvements in balance, upper body strength, lower body strength, flexibility, and physical function. Based on the findings by Allen et al. [60], the enhancements in physical health observed among the participants in this study could be attributed to older adults’ ability to perform dual activities with less difficulty due to practice. Additionally, psychomotor training seems to have favorable effects on the central nervous system, which could favor cognitive, psychological, and psychiatric treatments in people with MCI [61].

The combined training, which includes specific motor activities along with cognitive training, is likely to have produced beneficial effects on visuospatial activities such as gait. This type of training also reduces the risk of falls and enhances executive functions through the transfer of benefits to daily activities performed by older adults. In Bherer et al.’s study [62] comparing dual-task training effects in older and younger adults, older adults showed a greater improvement in dual-task transfer effects and costs compared to younger adults, which aligns with our findings.

It is noteworthy that the control group did not exhibit significant changes in any of the variables studied. Concerning cognitive variables, the enhancement of cognitive function in older adults with MCI or dementia can prove challenging due to individual factors such as dietary habits, supplementation, sleep quality, social interactions, and disease stage [63]. Nevertheless, the attenuation of the natural progression of the condition is regarded as a favorable outcome, as evidenced by the control group in this study. Despite not showing improvement, they did not experience deterioration. In relation to executive functions and verbal fluency, our results are congruent with the findings reported by Gómez-Soria et al. [64] in their meta-analysis. They observed a notable degree of heterogeneity among the studies included and did not find statistically significant changes following cognitive stimulation.

This study has some limitations that should be acknowledged. Firstly, the presence of only two groups (intervention/treatment and control group) makes it challenging to determine whether the observed effects post-intervention are specifically due to the cognitive or psychomotor interventions. Secondly, while there were no significant baseline differences between the groups regarding sex, the proportion of female participants was notably lower than that of male participants. Therefore, future studies should place emphasis on including a more balanced representation of both sexes to ensure comprehensive insights.

## Conclusions

Our findings indicate statistically significant improvements in balance, gait, upper and lower body strength, flexibility, physical function, cognitive function, cognitive impairment, verbal fluency, and executive functions in the treatment/training group compared to previous measurements and to a control group. These improvements, evidenced by statistical significance on several tests such as the Tinetti scale, indicate a potentially reduced risk of falls, as well as improvements in physical and cognitive abilities. Clinically, these results underscore the importance of integrated interventions that include physical exercise and cognitive training to address both physical and cognitive health in older adults, suggesting a promising therapeutic approach to mitigate age-related decline and improve quality of life.

## Data Availability

The data presented in this study are available on request from the corresponding author. The data are not publicly available because, due to the sensitive nature of the questions asked in this study, participants were assured raw data would remain confidential and would not be shared.

## Abbreviations

BMI: Body mass index
BST: Back scratch test
CG: Control group
CSRT: Chair sit-and-reach test
EG: Experimental group
MCI: Mild cognitive impairment
MMSE: Mini-mental state examination
MoCA: The Montreal Cognitive Assessment
TMT: Trail Making Test
TUG: Timed Up and Go
